# Evaluating and rethinking public health for the 21st century: Toward vulnerable population interventions

**DOI:** 10.3389/fpubh.2022.1033270

**Published:** 2022-10-28

**Authors:** Angeline Chatelan, Saman Khalatbari-Soltani

**Affiliations:** ^1^Institute of Social and Preventive Medicine (ISPM), University of Bern, Bern, Switzerland; ^2^Geneva School of Health Sciences, University of Applied Sciences and Arts Western Switzerland (HES-SO), Geneva, Switzerland; ^3^Faculty of Medicine and Health, The University of Sydney School of Public Health, Sydney, NSW, Australia; ^4^ARC Centre of Excellence in Population Aging Research (CEPAR), University of Sydney, Sydney, NSW, Australia

**Keywords:** public health policy, preventive interventions, individual-centered interventions, population-centered interventions, vulnerable population interventions

## Introduction

Public health preventive interventions aim to improve population health through two main approaches. Firstly, individual-centered interventions seek to change knowledge and behaviors of individuals identified as at high risk of disease. Secondly, population-centered interventions are delivered across the whole population, without prior detection of individuals at increased risk of disease (1). Population-centered interventions can address three types of health determinants: (i) the personal behaviors (e.g., mass media campaigns to improve diet), (ii) the physical environment (e.g., clean air and water policies), and (iii) the social and economic environment (e.g., safe housing provision). Despite the significant role of both individual- and population-centered approaches in improving population health during the last decades, health inequities between socially, culturally, or financially disadvantaged groups within populations are increasing, at least for some health outcomes (2). This is partly due to shortcomings of both individual- and population-centered approaches. Learning from modern public health history and given the health emergencies such as the COVID-19 pandemic, this commentary argues that 21st-century public health should mainly invest in vulnerable population interventions. This approach aims to decrease health inequities between socially defined groups and is a necessary complement to population-centered interventions.

## Learning from the history: Shortcomings of the individual- and population-approaches

**Figure 1:**
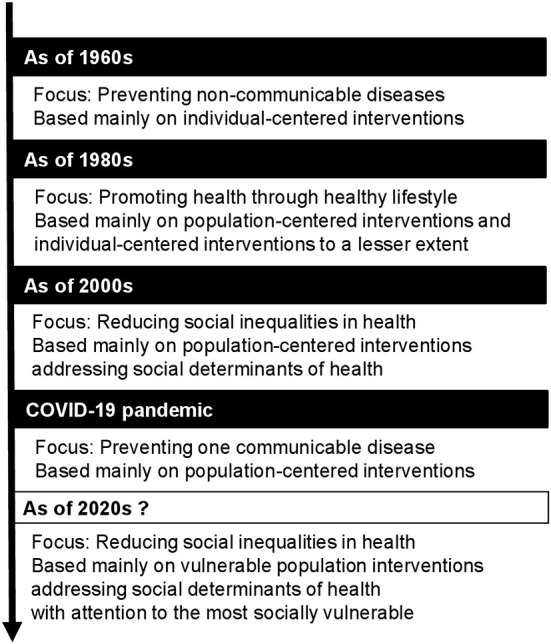
Overview of the modern history of preventive public health and its desirable future.

In the late 18th and 19th centuries, public health concentrated its efforts on improving sanitation and preventing communicable diseases using population-centered interventions (e.g., safe sewage disposal and mass vaccinations), which led to massive improvements in population health (3). For instance, in the United States, life expectancy at birth increased from <44 years in 1890 to more than 70 years in 1965 (79 years in 2020) (4). After the Second World War (Figure 1), non-communicable diseases (NCDs, e.g., cardiovascular disease, cancers) took over communicable diseases as the leading cause of death. Preventive public health interventions were primarily based on disseminating information to high-risk individuals regarding the risk of newly identified unhealthy behaviors (e.g., tobacco smoking, poor eating habits, low physical activity) (1).

The impact of this individual-centered approach to preventing NCDs was limited (1). An emblematic example was the Multiple Risk Factor Intervention Trial (MRFIT) involving 12,866 men at high risk of coronary heart disease (5). Despite intensive programs to decrease cardiovascular risk factors (i.e., stepped-care drug treatment for hypertension, smoking cessation program, fat-modified diet, and weight control when necessary), no significant differences in mortality rates were found between the intervention and control groups after seven years of follow-up (5).

One of the main critiques of individual-centered approaches has been its emphasis on framing the problem as one of personal responsibility. Providing psychoeducational health counseling regarding individual behavior modifications has been deemed insufficient in the absence of societal changes conducive to these changes (1). A second critique by Geoffrey Rose in the 1980s was that “a large number of people at a small risk may give rise to more cases of disease than the small number who are at a high risk” (1). Of note, it has been previously discussed that even if these approaches reduce the risk of those targeted, the persistence of the societal forces provides conditions for new people to enter the at-risk population (6).

Acknowledging that modifying individual behaviors without altering population-level life conditions is challenging and that lowering the mean level of risk (of disease) in everyone (rather than in high-risk individuals only) is more impactful, public health moved its focus away from disease prevention toward health promotion in the 1980s. Organized by the World Health Organization (WHO), the first International Conference on Health Promotion in 1986 in Ottawa established a Charter to achieve Health for all by the year 2000 and beyond. The Ottawa Charter represented a milestone for health promotion and stressed the critical role of environments, community, and public policy in promoting health in various sectors, such as legislation and fiscal measures (7). The Charter also defined health as “a resource for everyday life, and not the objective of living” and highlighted the importance of “enabling people to increase control over, and to improve, their health” (7). Health promotion concentrates on creating collective capacities for living mainly with population-centered interventions (e.g., smoking-free public spaces) rather than preventing disease at the individual level (e.g., smoking cessation programs).

Despite successes in the prevention and control of NCDs in different parts of the world through a variety of population-based interventions (e.g., smoke-free space, cigarette excise tax increase, tax on sugar-sweetened beverages), population-centered interventions have not accomplished their full potential. In the early 21st century, some public health experts noted the neglect of socially vulnerable groups (e.g., racial and ethnic minorities; socioeconomically disadvantaged groups) (6). For instance, population-level smoking rates have reduced, but social inequities in smoking have grown (8). These experts notably pointed out that population-centered approaches that address personal health behaviors and not the contextual conditions (fundamental causes) tend to widen social inequities in health (6). Indeed, less vulnerable individuals derive more benefits from the interventions than the most vulnerable, arguably due to the financial, cultural, and social resources available to each group (9, 10). For instance, women with higher incomes were more likely to be screened for cervical cancer screening than those with lower incomes in Ontario and the United States (11). Another example is the public information campaign for folate intake in women of childbearing age, which tended to be most effective among women with higher education (12).

In 2008, social inequities in health featured prominently in the WHO's report “Closing the Gap in a Generation: Health Equity Through Action on the Social Determinant of Health,” reflecting their global salience (2). This report called for health equity and argued that public health should focus on the social determinants of health, including gender, ethnicity, education, income (distribution), working conditions, access to sufficient healthy food, and housing (2). To achieve that, public (health) interventions should change the systems and organizations that shape the circumstances in which people grow, live, work, and age (2).

Then, in 2020, with the COVID-19 pandemic, population-centered interventions, such as social distancing, quarantine, mask-wearing, workplace closure, and vaccinations, have taken a front and center place. These population-centered interventions did not focus on social determinants of health, and as expected, benefits were limited among the most socially vulnerable. The latter were more exposed to the virus and were more likely to fall ill, die, and end up with long-haul COVID-19, further exacerbating health inequities (13, 14). Given the substantial inequities in COVID-19 and its outcome, few initiatives started focusing on vulnerable communities (e.g., the United States National Initiative to address COVID-19 health disparities among populations at high-risk and underserved, including racial and ethnic minority populations and rural communities) (15). However, these deliberate efforts are far behind the initial population-based efforts. The COVID-19 pandemic has thus highlighted again that socially vulnerable groups require different kinds of interventions.

## Future directions for preventive public health: Vulnerable population interventions

After the COVID-19 crisis and given other health emergencies such as climate change, it is the perfect time to rethink public health. Public health needs more vulnerable population interventions so that socially vulnerable groups are not left behind. If the past is any guide, future public (health) interventions should be population-centered and address the social determinants of health. Examples of these types of interventions are increased childcare institutions, strong and equal education systems, subsidized healthy school meals, safe housing provision, and a psychologically safe workplace. In addition, and according to the local needs, these population-centered interventions should be complemented with interventions targeted to the most socially vulnerable groups (6). Defined with local communities, these participatory interventions can be related to, for example, early childhood development programs, groceries with free foods, peer-support programs to quit smoking, and health literacy programs.

In the 21st century, preventive public health should invest more in a vulnerable population approach, i.e., population-centered interventions addressing the social determinants of health and combined with community-based participatory interventions when and where needed (6). This vulnerable population approach is the most likely to reduce health inequities and improve population health in the long term.

## Author contributions

AC and SK-S: conceptualization, writing—original draft, review, and editing. All authors contributed to the article and approved the submitted version.

## Funding

AC was supported by the Swiss National Science Foundation (Project No: 190277). SK-S was supported by the Australian Research Council Centre of Excellence in Population Ageing Research (Project No: CE170100005). The funders had no role in the preparation of the commentary and decision to publish.

## Conflict of interest

The authors declare that the research was conducted in the absence of any commercial or financial relationships that could be construed as a potential conflict of interest.

## Publisher's note

All claims expressed in this article are solely those of the authors and do not necessarily represent those of their affiliated organizations, or those of the publisher, the editors and the reviewers. Any product that may be evaluated in this article, or claim that may be made by its manufacturer, is not guaranteed or endorsed by the publisher.
